# A cross-sectional comparison of breastfeeding knowledge, attitudes, and perceived partners’ support among expectant couples in Mekelle, Ethiopia

**DOI:** 10.1186/s13006-020-00355-z

**Published:** 2021-01-04

**Authors:** Kidane Tadesse Gebremariam, Oksana Zelenko, Afework Mulugeta, Danielle Gallegos

**Affiliations:** 1 School of Exercise and Nutrition Sciences, Queensland University of Technology, Brisbane, Australia; 2 School of Public Health, College of Health Sciences, Mekelle University, Mekelle, Ethiopia; 3 School of Design, Queensland University of Technology, Brisbane, Australia

**Keywords:** Breastfeeding, Knowledge, Attitude, Support, Parents, Comparison

## Abstract

**Background:**

Breastfeeding is considered the biological norm and essential to reduce infant morbidity and mortality. Mothers are responsible for breastfeeding but the support of others, including their partners is an influential determinant. The aim of this study was to compare antenatal breastfeeding knowledge, attitudes, and perceived breastfeeding support of expectant couples in Mekelle, Ethiopia.

**Methods:**

As part of a randomised controlled trial of an mHealth intervention, 128 couples in their third trimester from three selected health centres in Mekelle city were recruited to participate between September and October 2018. Couples who each had a personal mobile phone, read and spoke Tigrigna, and lived together were included. Baseline data on breastfeeding knowledge, attitudes, and perceived support (breastfeeding savvy, help, appreciation, presence and responsiveness) were collected using previously validated tools through interview by health workers.

**Results:**

Compared to mothers, male partners were more likely to indicate their intention to provide breastfeeding appreciation (*p =* 0.02), breastfeeding presence (*p* = 0.002), and breastfeeding responsiveness (*p* = 0.04). The mothers’ prenatal perception of their partners’ intended breastfeeding support was lower than fathers’ perceptions to support their partners. Multiparous mothers had more positive perceptions regarding their partners’ breastfeeding savvy (*p* = 0.03), and breastfeeding help (*p* = 0.02) compared to primiparous mothers.

**Conclusions:**

Fathers in Ethiopia are potentially strong supporters of breastfeeding practice. Future breastfeeding interventions should promote the involvement of fathers in breastfeeding and encourage mothers to increase their partners’ involvement in breastfeeding.

## Background

Breastfeeding is the biological norm and the optimal mode for feeding infants to reduce morbidity and mortality [1]. Although more than 80% of neonates are “ever breastfed” globally, only 50% initiate breastfeeding (baby put to the breast within an hour of birth) [2, 3], and only one-third of infants are exclusively breastfed for the first 6 months of their life [2, 4, 5]. Suboptimal infant feeding practices, including prelacteal feeding (the feeding of an infant with something other than breastmilk in the first 3 days of life), non-exclusive breastfeeding, and use of artificial infant formula contribute to 1.4 million child deaths globally [6]. In Ethiopia, only 60% of babies are exclusively breastfed for the first 6 months [7]. While the Ethiopian government had set a goal of improving the level of exclusive breastfeeding to 70% by 2015, there has only been a 1 % improvement in exclusive breastfeeding rates over the last 5 years [8]. In Ethiopia, non-exclusive breastfeeding was the cause for significantly higher risks of neonatal [9] and infant [10] mortality, and acute malnutrition [11].

Breastfeeding is a complex biological, social and cultural practice influenced by a broad array of sociodemographic, biophysical and psychosocial factors [12]. Psychosocial factors, including maternal intention to breastfeed, breastfeeding self-efficacy, knowledge, attitude, and social support are associated with early initiation and continued exclusive breastfeeding [13, 14]. Studies from low-income countries have also shown the effects of psychosocial factors on maternal behaviour to exclusively breastfeed for 6 months and to continue breastfeeding for 2 years. These findings indicated that mothers with better breastfeeding self-efficacy are knowledgeable and have positive attitudes towards breastfeeding, and those who have access to support are more likely to exclusively breastfeed for 6 months [15, 16].

Modifiable factors, which impact maternal breastfeeding practices are primarily psychosocial components, including maternal intention to breastfeed, attitudes, self-efficacy and social support during lactation [13]. These psychosocial factors can be potentially modified through interventions focussed on breastfeeding education and promotion [13, 17].

Adequate support for lactating mothers is crucial to improve exclusive breastfeeding practice [3]. According to Dennis [18] unsupported mothers are less likely to initiate, and continue breastfeeding. Maternal breastfeeding intention is dependent on their partners’ breastfeeding attitude [19]. Mothers’ perception their partner’s preference about infant feeding affects their breastfeeding practice. Mothers continue breastfeeding when their partners prefer breastfeeding conversely, mothers cease breastfeeding when their partners prefer bottle feeding [19]. Maternal breastfeeding intention is strongly predicted by their partners’ breastfeeding beliefs, which impacts on their decision to breastfeed more than their own reasons [20]. Fathers can support their partners by providing breastfeeding information to motivate and assist them to breastfeed, as well as providing practical support with care of additional children and housework [21].

A majority of the studies in Ethiopia have assessed the effect of sociodemographic characteristics of mothers on breastfeeding exclusivity [22–25], with fewer addressing mothers’ knowledge attitudes and self-efficacy [16, 26]. A cross-sectional study from Ethiopia exploring involvement of fathers in breastfeeding has shown promising effects on breastfeeding practice. However, fathers’ participation was affected by the maternal perception about the role of the father in breastfeeding [27]. Works by non-government agencies in Ethiopia have identified a lack of knowledge and traditional gender roles as being limiting factors for fathers’ involvement in breastfeeding [28, 29]. Encouraging fathers’ involvement in breastfeeding, as well as the provision of breastfeeding information during antenatal care, would assist them to be more supportive during breastfeeding [30]. The understanding the level of breastfeeding knowledge, attitudes and the perception of partners’ support from the perspective of expectant couples would inform our understanding of effective breastfeeding interventions targeting fathers and mothers. Therefore, the aim of this study was to compare expectant couples’ breastfeeding knowledge, attitudes, and support in Mekelle, Ethiopia.

## Methods

### Study setting and design

This research took place in three health centres located in Mekelle, Ethiopia. Mekelle is a large city located in north Ethiopia. Mekelle has nine health centres, a tertiary hospital, and three general hospitals. This paper reports on baseline data from a quasi-experimental study, designed to test the effectiveness of a SMS-based breastfeeding education intervention targeting expectant couples attending antenatal care (ANC) in public health centres in Mekelle city, Ethiopia.

### Source and study population

Three health centres with the highest number of mothers attending ANC follow-up were purposively used as recruitment sites. A total of 293 mothers attending their antennal care (ANC) in these three public health centres were approached by nurses either in person at the health centre during the ANC appointment or via a phone call to check eligibility criteria. Eligible couples satisfied the following inclusion criteria: able to read and understand the local language (Tigrigna), living in a union, mother had no known medical issues that could hinder breastfeeding, there were no known issues with the foetus or pregnancy and both members of the couple were able to provide a written informed consent. Based on these criteria, 128 expectant couples were included in this study.

### Data collection

Data were collected through a face-to-face interview between September and October 2018 by trained nurses working in the health centre. A modified version of the cross-cultural adaption process was utilised, in which the questionnaires were translated to Tigrigna, after which the Tigrigna versions of the questionnaires were back-translated to English by two public health nutrition experts from Mekelle University [31]. Finally, face validity to check for understanding and language was conducted for all questionnaires with fathers and mothers who had children under 2 years of age but not reliability and validity assessment.

### Variables and measurement

Questions on father and mother characteristics such as age, educational status, income, and employment status; and pregnancy and childbirth-related variables such as parity, ANC provider, number of ANC appointments, breastfeeding information during ANC, breastfeeding experience, maternity leave provision, and breastfeeding intention were developed based on the literature [12, 13, 18, 22, 32].

Breastfeeding knowledge/awareness was assessed using a questionnaire adopted from the Food and Agricultural Organization (FAO) of the United Nations (UN) [33]. This questionnaire has ten open questions, which were later coded into Yes or No responses, based on the protocols. Each correct answer was scored, responses were totalled, and the percentage of correct responses recorded. Breastfeeding attitudes were measured using the Iowa Infant Feeding Attitude Scale (IIFAS). This tool has 17 questions, and uses a five-point Likert scale ranging from 1 = strong disagreement to 5 = strong agreement [34]. Out of the 17 questions nine were reverse scored, thus, these responses were recoded before calculating the total attitude score. The total score was calculated out of 85, with a minimum of 17 and maximum of 85. The questions are non-gendered and can be asked to men and women without modification.

The Partner Breastfeeding Influence Scale (PBIS) was used to measure perceived breastfeeding support and was assessed using five dimensions of partner breastfeeding support (Savvy (Cronbach alpha: men = 0.87, women = 0.82), helping (Cronbach alpha: men = 0.79, women = 0.82), appreciation (Cronbach alpha: men = 0.86, women = 0.84), breastfeeding presence (Cronbach alpha: men = 0.88, women = 0.82), and responsiveness (Cronbach alpha: men = 0.77, women = 0.76)). Mean scores were calculated from all scores, from 1 (extremely not supportive) to 5 (extremely supportive) for each breastfeeding support component [35].

### Data analysis

The data was analysed using IBM SPSS Statistics version 23 (IBM Crop. Released 2015. IBM SPSS Statistics for Windows, Version 23.0. Armonk, NY: IBM Crop). After data cleaning and coding, descriptive statistics were conducted. Sociodemographic characteristics were presented using frequency and percentage or mean with standard deviation. Normality tests were performed for each continuous variable using skewness test. Based on these tests further analyses were selected. Independent t-test or ANOVA, and Mann-Whitney U Test or Kruskal Wallis test were used for normal distribution and non-normally distributed data, respectively. Once the explanatory variables were fitted with the dependent variable (gender) variables with *p* - value < 0.05 were considered as having significant difference among mothers and fathers. Reporting follows the STROBE guidelines for cross-sectional studies.

## Results

### Sociodemographic characteristics

In this study 128 expectant couples participated. About half (46%) of the participants were first-time parents. Almost half (47%) of mothers did not have paid employment, and only a quarter (26%) of mothers and nearly a third (30%) of fathers had been educated beyond secondary school (Table 1).

**Table 1:** Sociodemographic and economic characteristics of fathers and mothers

Variables	Mothers *N* = 128	Fathers *N* = 128
Age (years)	Mean (26.8 + 4.7)	Mean (34 + 7.3)
Religion		
Orthodox Christian	121 (94.5%)	
Other	7 (5.5%)	
Educational Status		
Primary school	39 (30.4%)	42 (32.8%)
Secondary school	56 (43.8%)	48 (37.5%)
Tertiary	33 (25.8%)	38 (29.7%)
Household income	Mean (3629.5 + 2170.9)	
Employment		
No job	60 (46.9%)	14 (10.9%)
Own job	41 (32%)	67 (53.9%)
Employed	27 (21.1%)	47 (35.2%)
Number of Children		
0	58 (46%)	
1	35 (27%)	
2 and above	35 (27%)	

### Pregnancy and breastfeeding

Among the 128 expectant couples more than half 71 (56%) received their first antenatal care (ANC) for the current pregnancy at 4 to 6 months of gestation, and 73 (57%) of them made two or three ANC visits to the health centre. Among the 70 (55%) mothers who had previous breastfeeding experience, three-quarters (75%) had breastfed their last baby for more than 2 years. Almost all, 125 (98%), of mothers intended to exclusively breastfeed their babies for the first 6 months (Table 2).

**Table 2:** Pregnancy and previous breastfeeding experience of mothers

Variable	Frequency (%)
Months of pregnancy during first ANC visit	
1st to 3rd months	57 (44.5)
4th to 6th month	71 (55.5)
Place ANC received	
Health center	114 (89.1)
Other	14 (10.9)
ANC service provider for the current pregnancy	
Doctor	8 (6.3)
Midwife	80 (62.5)
Nurse	65 (50.8)
Health officer	20 (15.6)
Health extension worker	7 (5.5)
Number of ANC visits for the current pregnancy	
2–3	73 (57)
4 and above	55 (43)
Have you received breastfeeding information at ANC	
Yes	90 (70.3)
No	38 (29.7)
Do you have previous breastfeeding experience	
Yes	70 (54)
No	58 (46)
How long did you breastfeed your last child	
< 2 yrs	17 (24.6)
> = 2 yrs	52 (75.4)
Do you have maternity leave (employed mothers)	
Yes	25 (92.6)
No	2 (7.4)
How much maternity leave (employed mothers)	
1 Month	1 (3.8)
3 Months	6 (26.9)
4 Months	18 (69.3)
How do you intend to breastfeed your baby	
Breastmilk	125 (97.7)
Breastmilk + infant formula	3 (2.3)
How long should a baby be exclusively breastfed	
6 months	101 (79.9)
Others	27 (20.1)
Up to what age should a baby breastfeed	
Before 2 yrs	5 (3.9)
2 yrs. and above	123 (96.1)

### Breastfeeding knowledge, attitudes, and breastfeeding support of expectant couples

Table 3 presents the mean scores for breastfeeding knowledge, attitudes, and support. There were significant differences between fathers and mothers in the mean score for intention to discuss breastfeeding information (breastfeeding savvy), appreciation, and presence during breastfeeding, and responsiveness during breastfeeding. Comparing the intention to provide breastfeeding support, fathers had higher intention scores regarding supporting their partners compared to the mothers’ perception scores of the support their husband would provide (breastfeeding appreciation (*p* = 0.02), presence during breastfeeding (*p* = 0.002), and responsiveness during breastfeeding (*p =* 0.04)) (Table 3). One in five fathers (20%) and 13% of mothers had good breastfeeding knowledge, scores above 70%. In addition, mothers and fathers’ attitude score indicated neutral towards breastfeeding and formula-feeding (Table 4). Table 5 describes the differences in mean scores between multiparous and primiparous mothers in breastfeeding knowledge, attitudes, self-efficacy, and breastfeeding support. Multiparous mothers had better mean scores for breastfeeding savvy, breastfeeding helping, and breastfeeding appreciation perceptions with regard to their partners’ support, but there were no differences in the other variables compared to the primiparous mothers (Table 5).

**Table 3:** Breastfeeding knowledge, attitude, and support of expectant couples

Breastfeeding construct	Mothers	Fathers	*P* - value
Knowledge	Mean (61.5 + 14.4)	Mean (61.4 + 17.0)	0.66
Attitude	Mean (62 + 7.4)	Mean (61.4 + 8.5)	0.54
Support			
Savvy	Mean (36.4 + 7.6)	Mean (38 + 8)	0.05
Helping	Mean (29.0 + 5.4)	Mean (29.0 + 6.0)	0.15
Appreciation	Mean (24.9 + 4.9)	Mean (26 + 5.0)	0.02
Presence	Mean (24.4 + 4.8)	Mean (26 + 4.7)	0.002
Responsiveness	Mean (20 + 3.9)	Mean (21 + 4.0)	0.04

**Table 4:** Comparison of fathers’ and mothers’ knowledge of exclusive breastfeeding

Variables	Mothers Frequency (%)	Fathers Frequency (%)
First food for the newborn is breastmilk	125 (97.7)	126 (98.4)
Exclusive breastfeeding is giving the child breastmilk for the first 6 months	78 (60.9)	81 (63.3)
Babies should take only breastmilk for the first 6 months of their life	101 (78.9)	105 (82)
Breastmilk only is sufficient for the baby’s first 6 months of life	64 (50)	48 (37)
The baby should be breastfed on demand	22 (17.2)	32 (25)
Has knowledge on the benefits of exclusive breastfeeding to the baby	117 (91.4)	118 (92.2)
Exclusive breastfeeding is beneficial to the mother	48 (37.5)	53 (41.4)
Breastmilk supply can be sustained by having good nutrition/eating well	117 (91.4)	107 (83.6)
In times of absence the baby can continue to be exclusively breastfed by expressing breastmilk and storing	44 (34.4)	51 (39.8)
Health personnel can assist in overcoming breastfeeding difficulties	71 (55.5)	61 (47.7)
Knowledge category indicative of urgent intervention^a^ Good score (> 70%)	17 (13.3)	25 (19.5)

^a^According to FAO guideline score < =70% indicates urgent need for nutritional intervention

**Table 5:** Mean score difference between multiparous and primiparous mothers

Variables	Multiparous	Primiparous	*P* - value
Attitude	63	60.8	0.09
Knowledge	67.9	60.3	0.23
Self-efficacy	69.6	56.3	0.08
Savvy	70.9	66.8	**0.03**
Help	30	27.8	**0.02**
Appreciation	70.1	57.6	**0.05**
Presence	67.8	60.4	0.25
Responsiveness	20	19	0.69

## Discussion

The current study assessed the level of parents’ prenatal breastfeeding knowledge, attitudes, and perception of intended breastfeeding support. There are no known previous studies comparing these factors which affect exclusive breastfeeding practice, among fathers and mothers in Ethiopia. The current study showed that fathers had better intentions regarding showing appreciation of their partner’s breastfeeding; being present during breastfeeding or creating a pleasant environment for breastfeeding; and being responsive to breastfeeding with respect to being patient and understanding about the time it takes to breastfeed. Multiparous mothers had more positive perception with regard to their partners’ breastfeeding savvy, help and appreciation compared to primiparous mothers, but not in terms of breastfeeding knowledge, attitude, self-efficacy, presence and responsiveness. In addition mothers and fathers had low levels of breastfeeding knowledge [33].

The current study showed that there were no significant differences in breastfeeding knowledge scores of mothers and fathers. Parents’ knowledge about benefits of breastfeeding for the mother, breastfeeding on demand, and the importance of breastmilk expression were low. These scores were lower than maternal knowledge scores using the same tool in Ghana [36]. Many studies from Ethiopia indicate that women with better knowledge about the benefits of breastfeeding were more likely to exclusively breastfeed their infants for 6 months [11, 26, 32, 37, 38]. Previous study also indicated that fathers lack specific knowledge about infant and young infant feeding [28]. It has been suggested that fathers need more information related to breastfeeding and how they could support their partners [39]. Improving the understanding of optimal infant and child feeding could improve the likelihood of exclusive breastfeeding practice [40]. A systematic review from low- and middle-income countries also revealed that parents who received breastfeeding education to improve awareness were more likely to initiate breastfeeding early, exclusively breastfeed, and practice continued breastfeeding to at least 2 years of age [41].

Positive attitudes towards breastfeeding determines maternal exclusive breastfeeding practice. According to Dennis [18] and Meedya [13] mothers with positive attitudes towards breastfeeding are at lower risk of early discontinuation of breastfeeding. Positive attitude of mothers to breastfeeding substantially influences their exclusive breastfeeding practice by improving their prenatal exclusive breastfeeding intention [40]. Fathers’ attitude on whether their partners should breastfeed strongly predicts the level of maternal intention to breastfeed. Mothers who perceive that their partners prefer breastfeeding are less likely to cease breastfeeding at any time compared to those who perceive their partners prefer bottle feeding, or are indecisive about how their child is fed [19]. The current study showed that there was no significant difference in breastfeeding attitudes between mothers and fathers. Previous studies showed that maternal breastfeeding attitude was significantly correlated with the father’s breastfeeding attitude score, indicating parents share similar breastfeeding attitudes [42]. In line to the current study, previous study showed that breastfeeding attitude of fathers and mothers were almost similar [43].

Fathers believe encouraging and showing appreciation to their partner during breastfeeding is one way of providing support to mothers to improve breastfeeding practice [44]. The current study showed that fathers scored better regarding their intentions to encourage and value breastfeeding compared to the perception of mothers regarding their expectations of partner support. Multiparous women had higher expectations possibly based on previous experience. While such a difference is perhaps to be expected, the low expectations of mothers is something that could be addressed. Fathers indicated that they could help their partners by encouraging breastfeeding. According to the assessment of Alive & Thrive in the Tigray region, Ethiopian, fathers have a strong interest in improving their knowledge in relation to child feeding and have reflected their willingness to support their partners [29]. According to Tohotoa et al. [30] mothers believe that fathers make a difference in encouraging mothers to do the best thing with regard to breastfeeding. A previous study has shown that, mothers who perceived that their partners preferred breastfeeding were less likely to cease breastfeeding at any time compared to those who perceived their partners preferred bottle feeding, or were indecisive about how their child was fed [19]. Therefore, future interventions should emphasise the importance of encouragement and showing appreciation for breastfeeding among both fathers and mothers.

The fathers’ role in providing physical and emotional support to their breastfeeding partner could positively influence the mother’s breastfeeding practice. Mothers want fathers to be advocates for breastfeeding, and to show them their emotional support through their presence during breastfeeding [45]. The current study showed that, fathers believed they should be close to their partner during breastfeeding to improve maternal confidence in order to enhance continued breastfeeding. However, mothers’ perceptions of their partners’ support during breastfeeding was low. Previous studies showed that fathers’ presence during and support of improved breastfeeding [46], and reduce maternal anxiety and feelings of isolation [45]. Although fathers believed that breastfeeding support is important, their involvement is determined by their partners perception on the role they could play, and fathers are not part of breastfeeding decision, and most of the time fathers felt left out [47–49]. Therefore, it is important to inform fathers and mothers on how fathers can assist their partner during breastfeeding and be part of the decision-making around breastfeeding.

Responsiveness is an individual’s belief about how their partner understands, cares and validates their decisions [35]. According to the current study fathers’ perceptions regarding their responsiveness or the degree to which fathers were sensitive to the needs of their partners and respected their decisions was significantly higher in fathers compared to mothers. Again, this is to be expected and for mothers with previous experience based on this prior experience. Fathers’ breastfeeding responsiveness improves maternal breastfeeding satisfaction, helping mothers to comfortably breastfeed and to continue breastfeeding [35]. When fathers have positive attitude towards and the ability to provide the required breastfeeding support to their partner, they can bring a significant change to maternal breastfeeding practice [50]. The positive perception of fathers in the current study and whether they are able to provide the required support with regard to breastfeeding responsiveness indicates they could have a positive influence on breastfeeding practice. However, the low expectations of mothers regarding the support their partners could provide potentially hinders their involvement and the provision of this support.

One of the strengths of this study is that it is one of only a few studies comparing breastfeeding related knowledge, attitudes, and intended/expected support among expectant parents. As the data was collected during the antenatal period and therefore not reliant on retrospective memory. There are however a number of limitations. The data could be subject to social desirability bias, as the data were collected by trained nurses from the health centre attended by the couple. Although the tools went through face validity, but reliability and validity testes were not conducted in Ethiopian context. In addition, there were no indications of other support provided in the household that may have attenuated the expectation and intention to provide support. For example, the presence of a highly supportive grandparent may have influenced the support provided by the partner. Finally, the sample was limited in size and geographical location and therefore may not be representation of the general pregnant population in Ethiopia.

## Conclusions

Fathers tend to show better perceived breastfeeding support in terms of breastfeeding appreciation, presence, and responsiveness. The low perception of mothers with regard to fathers’ support could cause exclusion of fathers from breastfeeding and the decision regarding breastfeeding which could negatively influence breastfeeding practice. The breastfeeding knowledge score of mothers and fathers was low indicating the need for specific information. This low level of breastfeeding knowledge could affect parents’ breastfeeding attitudes; therefore, breastfeeding interventions should address breastfeeding knowledge, attitude, and fathers’ support.

## Data Availability

The datasets used and/or analysed during the current study are available from the corresponding author on reasonable request.
